# A Case of Metformin-Associated Lactic Acidosis

**DOI:** 10.7759/cureus.38222

**Published:** 2023-04-27

**Authors:** Rabia Mahmood, Daniel Maccourtney, Megha Vashi, Ayman Mohamed

**Affiliations:** 1 Internal Medicine, Ascension St. John Hospital, Detroit, USA; 2 Internal medicine, Ascension St. John Hospital, Detroit, USA

**Keywords:** diabetes mellitus type 2, anion-gap metabolic acidosis, metabolic encephalopathy, acute renal failure and hemodialysis in icu, metformin-associated lactic acidosis

## Abstract

Metformin is a US FDA-approved oral anti-hyperglycemic medication used to treat non-insulin-dependent diabetes mellitus (NIDDM). Metformin, a biguanide drug, works by reducing glucose production in the liver, decreasing intestinal absorption, and improving insulin sensitivity, leading to lower blood glucose levels. Metformin is generally considered to be a medication with a good safety profile and high tolerability. However, metformin therapy is associated with an uncommon but potentially serious complication known as metformin-associated lactic acidosis (MALA), which is marked by severe lactic acid accumulation in the bloodstream. This case introduces an elderly female with multiple comorbidities who presented with confusion, malaise, and lethargy. Her laboratory findings revealed acute renal failure, severe metabolic acidosis, and significantly elevated lactic acid levels consistent with sepsis and possibly MALA. Aggressive resuscitation with fluids and sodium bicarbonate was initiated. Antimicrobial drugs were started for urinary tract infections. She subsequently required endotracheal intubation with invasive ventilation, pressor support, and continuous renal replacement therapy. Her condition gradually improved over several days. The patient ultimately recovered, and at the time of discharge, metformin was discontinued, and a sodium-glucose cotransporter-2 (SGLT-2) inhibitor was initiated. This case underscores the relevance of MALA as a potential complication of metformin therapy, particularly in patients with underlying kidney disease or other risk factors. Timely detection and prompt management of MALA can prevent progression to a critical stage and thus avoid potentially fatal outcomes.

## Introduction

Metformin is a widely prescribed anti-hyperglycemic agent for non-insulin-dependent diabetes mellitus (NIDDM). It is an oral biguanide that inhibits gluconeogenesis, gastrointestinal absorption of glucose and improves insulin sensitivity [1]. Despite its therapeutic efficacy, metformin carries a black box warning for lactic acidosis, necessitating its immediate cessation in any patient suspected of developing this condition due to toxic metformin levels [2]. The uncommon but potentially fatal complications of metformin-associated lactic acidosis (MALA) are a concern for clinicians. While the exact pathophysiological mechanism remains unclear, it is believed that metformin's inhibition of mitochondrial respiration in the liver reduces glucose production and leads to a decrease in the energy supply required for gluconeogenesis. This energy deficit can result in an increase in lactate production, which, if not cleared adequately, can accumulate in the bloodstream [3,4]. Notably, the risk of developing this complication is highest in patients with pre-existing conditions, such as renal impairment or liver disease that interfere with lactate clearance. In cases of toxicity, the resultant lactic acidosis can lead to severe metabolic acidosis and substantial morbidity and mortality if not managed promptly and effectively [4]. Consequently, physicians should remain highly vigilant when monitoring patients on metformin therapy for any signs of toxicity and be equipped to intervene swiftly in the event of an adverse reaction. In this article, we narrate a scenario involving a 66-year-old female diagnosed with MALA, initially presenting with a range of non-specific symptoms. The management of this condition posed several significant challenges, which we explore in detail, highlighting the critical role of timely recognition and intervention in preventing adverse outcomes. Through this case study, we aim to underscore the significance of prompt diagnosis and treatment of MALA, with an emphasis on providing valuable insights into the identification, treatment, and prevention of this serious complication. 

## Case presentation

A 66-year-old Caucasian female presented to the ED with a chief complaint of confusion. The patient could only provide limited meaningful history; therefore, the majority of the history was obtained by her family members who were present at the bedside. Per the family, the patient was noted to be increasingly confused over the past 2-3 days. The patient's symptoms were associated with generalized weakness, malaise, and lethargy. She had decreased oral intake over the past day. Per the family, the patient had not complained of any fever, chills, chest pain, dyspnea, abdominal pain, nausea, vomiting, or diarrhea; however, she had endorsed a 3-4 day history of burning pain with urination. The patient's medical history was remarkable for essential hypertension, hyperlipidemia, NIDDM, cerebrovascular accident with residual right-sided deficits, and seizure disorder. On medication reconciliation, the patient was noted to be taking aspirin 81 mg daily, atorvastatin 40 mg daily, metformin 1000 mg twice a day, lisinopril 10 mg daily, and amlodipine 10 mg daily. Per family members, the patient was compliant with all of her home medications. The patient's social history was negative for alcohol or recreational drug use. On arrival at the ED, vital signs were significant for hypothermia at 35.5°C, heart rate of 120 beats per minute, and blood pressure of 141/77 mmHg. Physical examination revealed a lethargic elderly female who was oriented to her name but not to the place and time. Head and neck examination revealed dry mucous membranes without discoloration. Skin examination revealed reduced skin turgor. On the evaluation of the abdomen, the patient was noted to be tender to palpation of the suprapubic region. The remainder of her physical examination was unremarkable. Laboratory tests were ordered, and a complete blood count (CBC) revealed leukocytosis and normochromic normocytic anemia. A comprehensive metabolic panel (CMP) revealed hypoglycemia, elevated blood urea nitrogen, significantly elevated serum creatinine at 6.06 mg/dL (baseline 0.8 mg/dL one month prior), hyperkalemia, critically low bicarbonate level, and anion gap metabolic acidosis. Serum sodium and calcium level were within normal limits. The patient's lactic acid was severely elevated. Venous blood gas (VBG) revealed remarkably low pH. Table 1 shows her labs on admission. Her urinalysis was negative for ketones but showed positive nitrites and moderate leukocyte esterase. Rapid urine drug screen (RUDS) was negative. TSH with reflex to free T4, serum ammonia level, acetaminophen, and serum salicylate level were all within normal limits. Upon chart review, her hemoglobin A1c (HbA1c) from three months prior was elevated at 8.0%. Point of care urinary bladder scan did not reveal evidence of urinary retention. Imaging, including a CT scan of the patient's head, revealed no acute intracranial processes. CT of her abdomen pelvis revealed no acute intra-abdominal processes. Blood and urine cultures were sent for evaluation. The patient received three ampules of sodium bicarbonate and was started on a continuous bicarbonate drip. She received one ampule of dextrose 50% for hypoglycemia and was started on aggressive IV fluid resuscitation. She was started on antimicrobial therapy with vancomycin and cefepime. The patient was admitted to the medical ICU for sepsis secondary to UTI and acute renal failure. More importantly, the patient's clinical presentation was highly suspicious for MALA. Upon arrival at the medical ICU, the patient became increasingly unresponsive with a Glasgow Coma Scale (GCS) of <8 (normal >15). The patient required endotracheal intubation for airway protection. The patient subsequently became hypotensive and was started on vasoactive agents for distributive shock from sepsis and sedative medications. Vascular surgery was consulted for emergent vascular access. Nephrology was consulted for emergent hemodialysis, and the patient was placed on continuous renal replacement therapy on the day of admission. 

**Table 1:** Patient’s lab values on admission, day 2, day 3, and day 15 of the hospital course.

	On admission (Day 0)	Day 2	Day 3	On discharge (Day 15)
WBC count (K/µL)	16.9	11.62	10.14	8.48
Hemoglobin (gm/dL)	8.3	7.3	7.30	7.10
Blood glucose (mg/dL)	23.0	167.0	103.0	163.0
Blood urea nitrogen (mg/dL)	63.0	32.0	11.0	23.0
Creatinine (mg/dL)	6.06	2.89	1.06	0.63
Potassium (mmol/L)	5.5	3.9	3.9	4.0
Anion gap (mmol/L)	36.0	26.0	18.0	8.0
Bicarbonate (mmol/L)	<2.0	16.0	17.0	23.0
Lactic acid (mmol/L)	17.2	4.7	1.1	-
Venous pH	6.73	7.49	7.34	-
Venous pCO2 (mmHg)	14.0	22.0	32.0	-

Over the next 24 hours, the patient had an increasing vasopressor requirement, and a second vasoactive agent was introduced. On day 2 of admission, the bicarbonate, pH, and lactic acid levels significantly improved. The bicarbonate drip was gradually discontinued. On the fourth day of admission, the patient's blood pressure improved, and all vasoactive agents were ultimately discontinued. The patient required continuous renal replacement therapy for five days, and on day 6, her creatinine improved to 1.15 mg/dL. At this time, the patient had adequate urine output. The patient's antibiotics were de-escalated after blood cultures demonstrated no growth, urine cultures grew Escherichia coli susceptible to ceftriaxone, and antibiotics were discontinued after completing seven days of ceftriaxone for UTI. On day 9 of admission, the patient was successfully extubated after she gradually returned to her baseline mental status. Ultimately the patient was discharged to a subacute rehabilitation facility for critical illness myopathy. Upon discharge, the patient's metformin was discontinued, and she was started on an SGLT-2 inhibitor by her primary care provider in the outpatient setting. 

## Discussion

Metformin is an anti-hyperglycemic medication accepted by the US FDA for the treatment of NIDDM [5]. In adults, metformin is recommended as the first-choice agent for NIDDM by the American Diabetic Association (ADA) [6]. In addition to this FDA-approved indication, metformin is also used off-label for managing weight gain associated with second-generation antipsychotic medications, polycystic ovarian syndrome, maternal gestational diabetes, and pre-diabetes [7]. Metformin, a biguanide drug, works by reducing glucose production in the liver, decreasing intestinal absorption, and improving insulin sensitivity, leading to lower blood glucose levels [1,3]. Metformin is renally eliminated from the body. The optimal plasma concentration of metformin is <2 mcg/mL, while levels above 5 mcg/dL are concerning and may indicate excess ingestion, reduced medication clearance secondary to kidney dysfunction, reduced clearance of lactate secondary to liver dysfunction, or increased production of lactate [1-4]. Although generally considered safe and well-tolerated, up to 30% of patients undergoing therapy may complain of GI symptoms. Despite being a rare occurrence, metformin use carries the risk of fatal lactic acidosis, particularly in patients with liver and kidney impairment, with an incidence of 1 in 30,000 patients [4]. In the case of MALA, the drug inhibits mitochondrial respiration, leading to impaired cellular energy production and lactate accumulation. The excess lactate contributes to the decline in pH and the development of metabolic acidosis. This decrease in pH can cause non-specific signs and symptoms such as altered mental status, nausea, vomiting, tachypnea, weakness, malaise, and lethargy [3,4]. In severe cases, lactic acidosis can cause multi-organ failure and subsequent death. Risk factors for MALA include renal dysfunction, hepatic impairment, hypoxemia, sepsis, and alcohol abuse. These factors can reduce serum pH and/or interfere with properly eliminating lactic acid [4]. To prevent the development of MALA, metformin usage requires careful consideration in patients with pre-existing liver and/or renal impairment. The latest recommendations by the FDA regarding the use of metformin in patients with different degrees of renal dysfunction, based on their estimated glomerular filtration rate (eGFR), are as follows: For individuals whose eGFR is >45 mL/min/1.73 m^2, metformin is safe to use. Metformin is contraindicated for individuals whose eGFR is <30 mL/min/1.73 m^2. For individuals whose eGFR is between 30 and 45 mL/min/1.73 m^2, refrain from introducing metformin therapy. Healthcare professionals must reassess the risks and benefits of continuing therapy for individuals whose eGFR drops <45 mL/min/1.73 m^2 while on metformin therapy. The FDA also advises annual monitoring of eGFR for all metformin users [5].

The ADA recommends metformin use in individuals with milder forms of liver disease (i.e., non-alcoholic steatohepatitis and compensated cirrhosis). However, metformin should be used with caution in patients with moderate-to-severe liver disease (i.e., cirrhosis with impaired liver function). Furthermore, metformin is typically contraindicated in those with end-stage liver disease [6,7]. Decisions regarding the use of metformin in liver disease should be made, taking into consideration the individual patient's medical history, liver function, and overall health status. Dosage adjustments may be necessary based on the severity of renal dysfunction and liver disease. MALA is a diagnosis of exclusion and requires broad laboratory testing to rule out other causes of lactic acidosis and high anion gap metabolic acidosis. Initial tests should include a complete blood count, basic metabolic panel, hepatic panel, coagulation studies, arterial blood gas analysis, serum lactate, serum ethanol, serum acetaminophen, and serum salicylate levels. Ethylene glycol and methanol levels should also be obtained if there is suspicion of toxic alcohol ingestion, as these can also contribute to high anion gap metabolic acidosis. Obtaining metformin levels may not be immediately helpful in the diagnosis of MALA as these tests typically require a send-out to a reference lab, and results may not be readily available at the time of presentation [3,4]. Management of severe metabolic acidosis due to metformin toxicity involves aggressive treatment to correct the underlying metabolic disturbance. The management of metformin toxicity is largely supportive [8]. To date, the primary treatment of metformin toxicity is using renal replacement therapies. Metformin's lack of significant protein binding and relatively small molecular weight allows it to be efficiently removed from the bloodstream through hemodialysis and continuous renal replacement therapy [8,9]. In addition, underlying metabolic acidosis can be treated with sodium bicarbonate therapy [10]. Furthermore, concurrent issues such as hypovolemia should be addressed by ensuring adequate fluid resuscitation. Metformin carries a black box warning for lactic acidosis and should be discontinued immediately in all patients with suspected MALA. Severe metabolic acidosis can lead to significant morbidity and mortality if not managed promptly and effectively. Physicians should be vigilant in monitoring patients on metformin therapy for signs of toxicity and be prepared to intervene quickly in the event of an adverse reaction. 

## Conclusions

In conclusion, this case illustrates the potentially fatal complications of metformin therapy, especially in those with underlying renal impairment or other risk factors. In order to prevent fatal outcomes associated with MALA, prompt recognition and early intervention are crucial. Further research on metformin toxicity, specifically the mechanism of action underlying MALA, could provide valuable insights into the condition's prevention, diagnosis, and management. Research could also explore the relationship between metformin toxicity and other risk factors, such as renal and hepatic dysfunction, and identify additional risk factors that may increase the likelihood of developing MALA. Furthermore, it may be necessary to evaluate the current dosing recommendations for metformin and consider developing new guidelines to minimize the risk of toxicity in vulnerable patient populations. Improving understanding of MALA could lead to developing novel therapeutic approaches and diagnostic tools to prevent or manage this condition, ultimately improving patient outcomes.
